# Risk Factors of Extensively Drug Resistant Typhoid Fever Among Children in Karachi: Case-Control Study

**DOI:** 10.2196/27276

**Published:** 2021-05-11

**Authors:** Anum Vighio, Muhammad Asif Syed, Ishfaque Hussain, Syed Masroor Zia, Munaza Fatima, Naveed Masood, Ambreen Chaudry, Zakir Hussain, Mirza Zeeshan Iqbal Baig, Mirza Amir Baig, Aamer Ikram, Yousef S Khader

**Affiliations:** 1 Field Epidemiology and Laboratory Training Program Pakistan Islamabad Pakistan; 2 Department of Public Health, Community Medicine and Family Medicine Faculty of Medicine Jordan University of Science and Technology Irbid Jordan

**Keywords:** case-control study, drug resistance, extensively drug resistant typhoid fever, risk factors, typhoid fever

## Abstract

**Background:**

Extensively drug resistant typhoid fever (XDR-TF) has been responsible for an ongoing outbreak in Pakistan, which began in November 2016.

**Objective:**

This study aimed to determine the risk factors associated with XDR-TF.

**Methods:**

This age- and sex-matched case-control study was conducted during May-October 2018 in Karachi. All patients with XDR-TF were identified from the laboratory-based surveillance system data. Cases included patients aged <15 years living in Karachi with culture-positive *Salmonella enterica* serovar Typhi with resistance to chloramphenicol, ampicillin, trimethoprim/sulfamethoxazole, fluoroquinolones, and third-generation cephalosporins. Age- and sex-matched controls included children free from the symptoms of TF, aged under 15 years, and residing in Karachi. All controls were recruited from among those who attended outpatient clinics.

**Results:**

A total of 75 cases and 75 controls were included in this study. On univariate analysis, the odds of having XDR-TF were 13-fold higher among participants who used piped municipal water than among those who did not (odds ratio [OR] 12.6, 95% CI 4.1-38.6). The use of bore water was significantly associated with XDR-TF (OR 5.1, 95% CI 1.4-19.0). Cases were more likely to report eating French fries with sauce (OR 13.5, 95% CI 3.9-47.0) and poppadum (OR 3.4, 95% CI 1.7-6.7) from street vendors than controls. Boiling water at home was negatively associated with XDR-TF (OR 0.3, 95% CI 0.2-0.7). On multivariate analysis, 2 factors were independently associated with XDR-TF. Using piped municipal water (OR 10.3, 95% CI 3.4-30.4) and eating French fries with sauce from street vendors (OR 8.8, 95% CI 2.1-36.2) were significantly associated with an increased odds of XDR-TF.

**Conclusions:**

Community water supply and street food eating habits were implicated in the spread of the superbug *S* typhi outbreak, which continues to grow in Karachi. Therefore, it is recommended to improve the community water supply to meet recommended standards and to develop a policy to improve the safety of street food. In addition, health authorities are required to conduct mass vaccination for TF among high-risk groups.

## Introduction

Typhoid fever is a preventable disease caused by *Salmonella enterica* serovar Typhi (*S* Typhi). A systematic review estimated an occurrence of 26.9 million culture-confirmed cases annually [1]. Typhoid fever affects high-risk populations in low- and middle-income countries [2]. Vaccination, access to clean water, and improved sanitation are effective means to prevent the spread of typhoid.

Antibiotics are vital to the treatment of typhoid. However, antibiotic-resistant pathogens have become increasingly prevalent [3]. Antibiotic resistance is an emerging public health threat worldwide. *S* Typhi resistant to first-line antibiotics (ampicillin, trimethoprim/sulfamethoxazole, and chloramphenicol) was initially considered multidrug-resistant. Reduced susceptibility to second-line drugs (ie, ﬂuoroquinolones) has also been widely reported since these had then emerged as the preferred treatment for multidrug-resistant typhoid fever. Ceftriaxone and azithromycin are now used to treat patients with typhoid fever, who are nonresponsive to ﬂuoroquinolones and first-line drug treatments [4].

In November 2016, the first ever recorded outbreak of extensively drug resistant typhoid fever (XDR-TF) (characterized by resistance to ampicillin, trimethoprim/sulfamethoxazole, chloramphenicol, ﬂuoroquinolones, and ceftriaxone) was identified in the Sindh Province in Pakistan. Afterward, increased numbers of XDR-TF cases have been reported from different parts of the country, which were associated with abysmal sewage and water systems, coupled with low vaccination rates and overpopulated city dwellings [5]. Sporadic cases of XDR-TF have been reported worldwide, thus raising the fear of antibiotic failure at a global level [5].

A robust laboratory-based surveillance system for XDR-TF was established in Karachi (Sindh Province) in December 2018. This study aimed to determine factors associated with XDR-TF among children under 15 years of age in Karachi.

## Methods

### Study Design and Setting

This age- and sex-matched case-control study (75 cases and 75 controls) was conducted during May-October 2018 in Karachi, the capital city of Sindh Province in Pakistan. All patients with XDR-TF were identified from the laboratory-based surveillance system data through simple random sampling. If the patient provided incomplete information or did not agree to participate, another case was chosen at random. Cases included patients with culture-positive *S* Typhi resistant to chloramphenicol, ampicillin, trimethoprim-sulfamethoxazole, fluoroquinolones, and third-generation cephalosporins, who are aged <15 years and living in Karachi. Cases aged over 15 years and those who are not permanent residents of Karachi were excluded. Age- and sex-matched controls included children who are free from the symptoms of typhoid fever, aged under 15 years, and residents of Karachi. Those who had experienced fever within the past 1 month were excluded. All controls were recruited from among those who attended outpatient clinics. This study was conducted after the formal approval of the Director General Health Services, Government of Sindh, and ethical approval was obtained from the ethics review board of Mohammad Medical College, Sindh. Verbal consent was obtained from the guardians or parents of the participants before obtaining data, and patient confidentiality was maintained by allotting a serial number to each participant.

### Data Collection

For both cases and controls, face-to-face interviews were conducted with caregivers or parents, using a standardized questionnaire. The questionnaire collected information about the participants’ demographic and clinical characteristics and modifiable risk factors. For cases, information was sought for a period of exposure from 3 weeks prior to the onset of symptoms; for controls, information was sought for a period from 3 weeks prior to the date of the interview. Other questions included those related to sources of drinking water, boiling of water for domestic use, and street food eating habits among children, including the consumption of poppadum, French fries served by default with homemade sauce, and ice cream.

### Environmental Samples

Water samples were obtained from the houses of infected cases and sent to the Pakistan Council of Research in Water Resources (Karachi). All samples were tested for the presence of fecal coliform bacteria, turbidity, and pH. Samples were not obtained from the controls owing to the lack of resources.

### Statistical Analysis

Data are expressed as means and percentages. Univariate and multivariate analyses were conducted using binary logistic regression to determine factors associated with XDR-TF. Odds ratios (ORs) and their corresponding 95% CI values were reported. A *P* value of *<*.05 was considered significant. Epi Info (version 7, Centers for Disease Control and Prevention) was used for statistical analysis. 

## Results

### Participants’ Characteristics

A total of 75 cases and 75 controls were included in this study. Among cases, males were predominant (n=40, 53%). Almost half (n=34, 45%) of the cases aged 5-9 years (mean 6.5 years, range 1-177 months). The majority of cases (97%) and controls (96%) were not vaccinated against typhoid (Table 1).

**Table 1:** The characteristics of 75 cases of extensively drug resistant typhoid fever and 75 age- and sex-matched controls in Karachi, Pakistan, in 2018.

Characteristics		Cases (n=75), n (%)	Controls (n=75), n (%)
**Gender**			
	Male	40 (53)	40 (53)
	Female	35 (47)	35 (47)
**Age (years)**			
	0-4	27 (36)	27 (36)
	5-9	34 (45)	34 (45)
	10-14	14 (19)	14 (19)
**Household income**			
	<US $125	60 (80)	50 (67)
	≥US $125	15 (20)	25 (33)
**Vaccinated for typhoid**			
	Yes	2 (3)	3 (4)
	No	73 (97)	72 (96)
**Residential area**			
	Urban	54 (72)	40 (53)
	Rural	21 (28)	35 (47)

### Univariate Analysis

On univariate analysis, the odds of having XDR-TF was 13-fold higher among participants who used piped municipal water than among those who did not (OR 12.6, 95% CI 4.1-38.6). The use of bore water was significantly associated with XDR-TF (OR 5.1, 95% CI 1.4-19.0). Cases were more likely to report eating French fries with sauce (OR 13.5, 95% CI 3.9-47.0) and poppadum (OR 3.4, 95% CI 1.7-6.7) from street vendors than controls. Boiling water at home was negatively associated with XDR-TF (OR 0.3, 95% CI 0.2-0.7) (Table 2).

**Table 2:** Univariate analysis of risk factors among 75 cases of extensively drug resistant typhoid fever and 75 age- and sex-matched controls enrolled in this study in Karachi, Pakistan, in 2018.

Factors		Odds ratio (95% CI)	*P* value
**Drinking water source**			
	Bottled water	Ref^a^	N/A^b^
	Piped municipal water	12.6 (4.1-38.6)	<.001
	Bore water	5.1 (1.4-19.0)	.01
	Tanker water	3.8 (0.8-17.6)	.08
	Water treatment: boiling drinking water (yes vs no)	0.3 (0.2-0.7)	.003
**Common street food eating habits**			
	French fries with sauce (yes vs no)	13.5 (3.9-47.0)	<.001
	Ice cream (yes vs no)	2.3 (1.0-5.1)	.05
	Poppadum (yes vs no)	3.4 (1.7-6.7)	<.001
Vaccination against typhoid (yes vs no)		0.7 (0.1-4.1)	.65

^a^Ref: reference for comparison.

^b^N/A: not applicable.

### Multivariate Analysis

On multivariate analysis, 2 factors were independently associated with XDR-TF. Using piped municipal water (OR 10.3, 95% CI 3.4-30.4) and eating French fries with sauce from street vendors (OR 8.8, 95% CI 2.1-36.2) were significantly associated with increased odds of XDR-TF.

### Environmental Samples

Of the 75 water samples collected, 51 (68%) showed the presence of fecal coliform bacteria.

## Discussion

### Principal Findings

Previous studies, 2 of which were conducted in Pakistan, have suggested that children under 15 years of age are more likely to be affected by typhoid fever in endemic countries than those who are older than 15 years [6-9]. In this study, numerous cases were predominantly children aged under 15 years. This may be associated with high-risk eating behavior, poor hygiene practices in school and community settings, and lower immunity [10,11]. However, some studies have reported contrary findings and showed that the more likely affected age group involved those aged over 15 years [12,13].

Most of the XDR-TF cases were males, which is concurrent with previous reports from Pakistan and Uganda [7,12]. Although some studies found females to be predominant [13,14], which may be expected owing to different cultural and social norms in different countries.

Socioeconomic status has been discussed in the fundamental cause theory of health inequalities, stating it to be a risk factor for deteriorated health outcomes over time [15,16]. Furthermore, a low socioeconomic status is associated with higher odds of TF [17,18]. In this study, most cases belonged to a lower social background. Lower socioeconomic groups usually consume unsafe drinking water, are unable to purchase vaccines (because the typhoid vaccine is not included in the routine immunization schedule of Pakistan), have poor access to health care, and have less knowledge about the disease [19,20].

Urban regions of Karachi are highly populated and face multiple problems such as a poor drainage system that contaminates the water supply, poor garbage disposal, and environmental and sanitation problems. In this study, more cases were reported from the urban areas of Karachi. However, some studies have reported an even distribution of cases in urban and rural areas [21].

In this study, 3% of cases and 4% of controls were found to be vaccinated against TF. The low prevalence of vaccination is attributed to the high cost of the vaccine and absence of the vaccine in the Expanded Programme on Immunization. This small number of vaccinated individuals deterred us from establishing a relationship between a lower vaccination status and XDR-TF. A systematic review reported that typhoid vaccines are efficacious for the prevention of TF [22].

The use of piped municipal water was associated with increased odds of XDR-TF. Laboratory analysis of water samples revealed the presence of fecal coliform bacteria in the water supply in towns in Karachi, which indicates fecal contamination of water, which in turn suggests that the water supply may harbor *S* Typhi along with other pathogens transmitted through the fecal-oral route [23,24]. A meta-analysis reported that the risk of TF is 2-fold when using unsafe drinking water [17]. Furthermore, 2 outbreak investigation studies reported that contaminated or untreated water was the main contributor to the outbreak [12,21]. The other factor associated with the outbreak was the street food eating habits of children. In line with this finding, beverages from street vendors have also been implicated in an outbreak in Uganda [12].

### Limitations

One of the limitations of this study is that we were unable to acquire food samples from street food vendors. Future studies should include food sampling as well. We were able to detect fecal coliform bacteria in the water samples but were unable to isolate *S* Typhi because this facility is not available in water testing laboratories in Karachi.

### Conclusions

Community water supply and street food eating habits are implicated in the spread of the XDR-TF outbreak, which continues to grow in Karachi. Therefore, it is recommended to improve community water supply to meet recommended standards and to develop a policy to improve the safety of street food. In addition, health authorities should conduct mass vaccination against TF for high-risk groups.

## Abbreviations

OR: odds ratio
XDR-TF: extensively drug resistant typhoid fever
